# Fixação de lesões osteocondrais do joelho com palitos ósseos autólogos: Avaliação funcional de uma série de casos *

**DOI:** 10.1055/s-0046-1822638

**Published:** 2026-06-08

**Authors:** Luis Henrique Longo, Anna Carolina Pavelec Costa, Camille Midori Okuyama, Edmar Stieven Filho, Marcos Paulo Tercziany Vanzin, Paulo Victor Oliveira Vieira de Souza

**Affiliations:** 1 Serviço de Cirurgia do Joelho, Complexo Hospitalar do Trabalhador, Curitiba, PR, Brasil; 2 Hospital Universitário Evangélico Mackenzie, Curitiba, PR, Brasil; 3 Hospital de Clínicas da Universidade Federal do Paraná, Curitiba, PR, Brasil

**Keywords:** cartilagem articular, osteoartrite do joelho, transplante autólogo, cartilage, articular, osteoarthritis, knee, transplantation, autologous

## Abstract

**Objetivo:**

Avaliar os desfechos clínico-funcionais de pacientes submetidos à fixação de fraturas osteocondrais traumáticas do joelho com palitos ósseos autólogos.

**Métodos:**

Realizamos um estudo retrospectivo transversal, que incluiu 11 pacientes operados entre 2021 e 2024. Foram analisados dados clínicos, radiográficos e funcionais, com aplicação do Formulário de Avaliação Subjetiva do Joelho do International Knee Documentation Committee (IKDC) durante o acompanhamento ambulatorial médio de 33,9 ± 10,6 meses.

**Resultados:**

A amostra apresentou média etária de 20,3 ± 5,4 anos, com predomínio de lesões patelares (81,8%). A avaliação radiográfica demonstrou consolidação do fragmento osteocondral em todos os casos, sem sinais de deslocamento ou falha da fixação. A pontuação média no IKDC foi de 75,0 ± 17,1. Não foram observadas complicações graves ou necessidade de reintervenções cirúrgicas.

**Conclusão:**

A fixação de fraturas osteocondrais traumáticas do joelho com palitos ósseos autólogos resultou em consolidação adequada e desfechos funcionais satisfatórios nesta série de casos, e configura-se como alternativa biológica segura, eficaz e de baixo custo para o manejo dessas lesões.

## Abstract (en)

**Objective:**

To evaluate the clinical and functional outcomes of patients who underwent fixation of traumatic osteochondral knee fractures using autologous bone pegs.

**Methods:**

We conducted a retrospective cross-sectional study including 11 patients who underwent surgery between 2021 and 2024. Clinical, radiographic, and functional outcomes were evaluated during a mean follow-up of 33.9 ± 10.6 months, including functional assessment using the International Knee Documentation Committee (IKDC) Subjective Knee Form.

**Results:**

The mean patient age was of 20.3 ± 5.4 years, with a predominance of patellar lesions (81,8%). The radiographic evaluation demonstrated consolidation of the osteochondral fragment in all cases, without signs of displacement or fixation failure. The mean IKDC score was of 75.0 ± 17.1 points. There were no major complications or reoperations.

**Conclusion:**

Fixation of traumatic osteochondral knee fractures with autologous bone pegs resulted in adequate consolidation and satisfactory functional outcomes in this case series, representing a safe, effective, and low-cost biological alternative to manage these lesions.

## Introdução

As fraturas osteocondrais da patela e do côndilo femoral, embora incomuns, 1 representam um desafio terapêutico, devido à capacidade limitada de cicatrização. 2 Essas lesões ocorrem predominantemente em indivíduos jovens e fisicamente ativos, e geralmente estão associadas a traumas agudos – sobretudo luxações patelares – ou a condições secundárias, como a osteocondrite dissecante. 3 O manejo ideal dessas fraturas ainda é motivo de debate na literatura, 2 4 uma vez que não há consenso sobre a técnica cirúrgica mais eficaz. No entanto, a preservação da cartilagem articular continua norteando o objetivo terapêutico, visto que a sua deterioração ou a formação de corpos livres pode levar a complicações graves, como limitação funcional da articulação e osteoartrose precoce. 2 5 6

Entre as técnicas cirúrgicas descritas para a fixação de fragmentos osteocondrais, a utilização de palitos ósseos autólogos tem se mostrado uma alternativa biologicamente vantajosa. 2 7 8 9 10 Essa abordagem, inicialmente descrita na literatura por Bandi e Allgoewer 11 em 1959, consiste no uso de enxertos ósseos retirados da metáfise tibial ipsilateral, que são inseridos no fragmento osteocondral para promover sua estabilização e integração óssea.

Por se tratar de tecido autólogo, o uso dos palitos ósseos favorece a integração biológica com o leito receptor, o que elimina a necessidade de implantes sintéticos. Essa técnica também dispensa o uso de materiais externos, o que reduz o risco de reações inflamatórias. 2 7 10 Em contrapartida, os métodos que utilizam parafusos metálicos frequentemente requerem remoção cirúrgica subsequente e podem ocasionar dano à cartilagem adjacente. 1 12 13 Como alternativa, os implantes bioabsorvíveis têm sido empregados para a fixação de fragmentos osteocondrais; contudo, seu uso permanece limitado e pode estar associado a reações de corpo estranho. 4 14 15 16

Apesar dos benefícios descritos, há escassez de estudos clínicos 2 17 que avaliem a funcionalidade em longo prazo de pacientes tratados com palitos ósseos autólogos. Diante disso, este estudo teve como objetivo avaliar os desfechos clínico-funcionais de pacientes com fraturas osteocondrais traumáticas do joelho tratados com essa técnica.

## Materiais e Métodos

Este estudo retrospectivo e transversal foi realizado em um hospital de referência em trauma, e foram avaliados pacientes submetidos à fixação de fraturas osteocondrais do joelho por meio de redução aberta e fixação interna com palitos ósseos autólogos, entre janeiro de 2021 e janeiro de 2024. O projeto foi aprovado no Comitê de Ética em Pesquisa da nossa instituição sob o protocolo CAAE: 87552425.7.0000.5225.

Foram identificados pacientes submetidos à fixação de lesões osteocondrais do joelho com palitos ósseos autólogos entre janeiro de 2021 e janeiro de 2024. Os critérios de inclusão foram: diagnóstico de fratura osteocondral traumática do joelho com fragmento passível de fixação, acompanhamento ambulatorial mínimo de 12 meses e maturidade esquelética confirmada por radiografias, com fises fechadas.

Os critérios de exclusão foram: histórico de cirurgia no joelho acometido, imaturidade esquelética, fragmentos cominutos ou inviáveis para fixação, além de lesões osteocondrais não traumáticas (como a osteocondrite dissecante).

Inicialmente, 13 pacientes foram considerados elegíveis. Um paciente foi excluído pelo histórico de cirurgia no joelho acometido, e outro, por apresentar diagnóstico de osteocondrite dissecante, num total de 11 pacientes incluídos na análise final.

No pré-operatório, todos os pacientes foram submetidos à avaliação clínica, radiografias, tomografia computadorizada (TC) e ressonância magnética (RM). O diagnóstico de fratura osteocondral traumática foi estabelecido pela identificação de fragmento deslocado, com envolvimento do osso subcondral, confirmado pela TC e pela RM, o que permitiu a diferenciação em relação às lesões puramente condrais, que não apresentam componente ósseo associado.

A TC foi utilizada principalmente para a avaliação da morfologia, do tamanho e da viabilidade do fragmento ósseo, ao passo que a RM permitiu a análise da cartilagem articular, do osso subcondral e das lesões associadas. Após essa etapa, os pacientes foram encaminhados ao procedimento cirúrgico, realizado em todos os casos pelo mesmo cirurgião especialista em cirurgia do joelho.

Os dados foram obtidos por meio da análise retrospectiva de prontuários eletrônicos e de consultas presenciais no ambulatório, conduzidas pelos demais membros da equipe. As variáveis analisadas incluíram: idade, sexo, lateralidade, etiologia da lesão, tamanho da lesão, topografia da fratura, dias transcorridos entre a lesão e o procedimento cirúrgico, número de palitos ósseos utilizados, tempo decorrido até a avaliação pós-operatória, presença de complicações e desfecho funcional segundo o Formulário de Avaliação Subjetiva do Joelho do International Knee Documentation Committee (IKDC). 18 Devido à natureza aguda dos casos, a pontuação no IKDC não foi sistematicamente coletada no pré-operatório, o que configura uma limitação deste estudo.

**Fig. 1:**
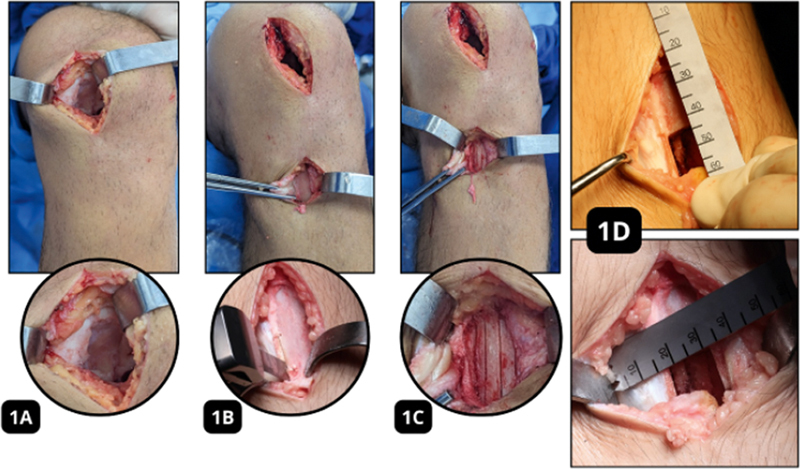
Sequência de exposição da lesão osteocondral e segmentação da cortical óssea metafisária anteromedial proximal.

Todos os procedimentos foram iniciados com inspeção artroscópica, o que permitiu a identificação e a ressecção cuidadosa do fragmento osteocondral. Em seguida, o leito da lesão foi exposto por meio de acesso parapatelar medial ou lateral, conforme a localização da fratura, para possibilitar a visualização direta da área acometida ( Fig. 1 ).

Com o auxílio de uma serra delicada, destacou-se um segmento da metáfise proximal anteromedial da tíbia ipsilateral, medindo aproximadamente 20 mm de comprimento por 15 a 16 mm de largura. Nesse fragmento, inicialmente foram realizados cortes longitudinais paralelos e equidistantes, que dividiram a cortical óssea anteromedial em segmentos regulares de cerca de 20 × 4 mm, o que facilitou a posterior confecção dos palitos ( Fig. 1 ).

**Fig. 2:**
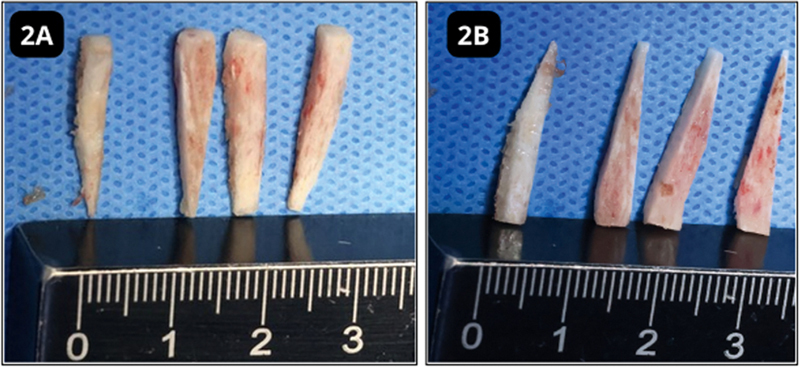
Palitos ósseos confeccionados.

Em seguida, os palitos foram refinados em mesa cirúrgica com o auxílio de osteótomos ou bisturi, e foram obtidos enxertos cilíndricos em formato de flecha, com dimensões aproximadas de 20 mm de comprimento, 2 a 3 mm de diâmetro na ponta, e 3 a 4 mm na base, adequados para fixação *press-fit* no leito da lesão( Fig. 2 ).

Com o leito cruentizado e o fragmento osteocondral reposicionado, realizou-se estabilização provisória com fio de Kirschner. Posteriormente, foi feita a pré-perfuração com broca delicada ou fio de Kirschner de 2,0 mm. A implantação dos palitos ósseos foi realizada por meio de impactação sob pressão ( *press-fit* ) através dos orifícios, utilizando martelo cirúrgico associado a *impactor* de enxerto, empregando-se no mínimo três palitos ósseos por lesão, até que a sua extremidade ficasse rente ao osso subcondral, sem protrusão na superfície articular. A sequência de fixação consistiu na implantação inicial do palito central, seguida de fixação periférica, buscando distribuição equidistante para garantir estabilidade rotacional e axial ( Fig, 3 ).

**Fig. 3:**
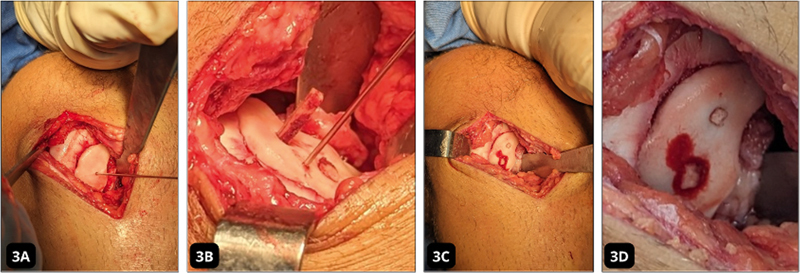
Fixação de lesão osteocondral com palitos ósseos e seu aspecto final.

Procedimentos associados, como a reconstrução do ligamento patelofemoral medial (LPFM), foram indicados em pacientes que apresentavam instabilidade patelar objetiva (luxação prévia documentada) associada à fratura.

No pós-operatório, os joelhos foram imobilizados com *brace* inguinomaleolar, mantido integralmente nas duas primeiras semanas. A mobilização passiva do joelho foi iniciada após esse período, inicialmente limitada à amplitude de 0 a 60°, com progressão gradual até a flexoextensão completa entre a quarta e a sexta semanas. Nos casos de lesões condilares femorais, recomendou-se suspensão completa da descarga de peso por 6 semanas, seguida de progressão gradual até apoio total na 12ª semana. Pacientes com lesões na patela permaneceram sem apoio de peso por 2 semanas. O retorno às atividades de vida diária foi autorizado de forma gradual após cerca de 3 meses, conforme a evolução clínica individual. A consolidação da fratura osteocondral foi definida por meio de exames radiográficos seriados, considerando ausência de linha de fratura visível e integração do fragmento ao leito ósseo em pelo menos duas incidências. A realização de RM de controle não foi um procedimento de rotina, sendo reservada para pacientes com evolução atípica.

Os pacientes foram acompanhados ambulatorialmente de forma regular, conforme o protocolo institucional. A alta ocorreu em torno de 12 meses de pós-operatório. Para este estudo, pacientes operados em períodos anteriores foram convocados a retornar ao ambulatório para reavaliação, ocasião em que receberam informações detalhadas sobre a pesquisa e assinaram o Termo de Consentimento Livre e Esclarecido (TCLE). Nessas consultas, o formulário do IKDC foi aplicado em momento único para cada paciente, não necessariamente coincidente entre os indivíduos, caracterizando, assim, uma análise transversal.

Os dados foram organizados em planilhas eletrônicas (Microsoft Excel, Microsoft Corp.) e analisados de forma descritiva. As variáveis quantitativas foram expressas em termos de calores de média, desvio-padrão ou mediana, conforme a distribuição. As variáveis categóricas foram expressas em termos de frequências absolutas e relativas.

## Resultados

Foram avaliados 11 pacientes submetidos à fixação de fraturas osteocondrais traumáticas do joelho com palitos ósseos autólogos, com idade média de 20,3 ± 5,4 anos. A maioria dos pacientes era do sexo masculino (63,6%; n = 7), e o joelho mais acometido foi o esquerdo (63,6%, n = 7). Quanto à etiologia, 8 pacientes (72,7%) apresentavam instabilidade patelofemoral (IPFM), ao passo que 3 pacientes (27,3%) tiveram fraturas decorrentes de traumas diretos.

A topografia da fixação foi predominantemente a patela (n = 9; 81,8%), seguida do côndilo femoral lateral (n = 2; 18,2%). O tamanho médio das lesões osteocondrais foi de 3,4 ± 1,1 cm ^2^ , com a utilização de uma média de 4,1 ± 1,6 palitos ósseos por procedimento. Em 3 casos (27,3%), foram realizados procedimentos cirúrgicos adicionais no mesmo tempo operatório, sendo 3 reconstruções do LPFM e 1 *release* do retináculo lateral.

O tempo médio decorrido entre a lesão e o procedimento cirúrgico foi de 3,5 ± 1,0 dias. O tempo médio de acompanhamento pós-operatório até a avaliação final foi de 33,9 ± 10,6 meses. Durante esse período, 3 complicações foram registradas: 2 pacientes apresentaram rigidez articular, e necessitaram de manipulação sob sedação em centro cirúrgico para a restauração da amplitude de movimento, e 1 paciente evoluiu com síndrome de dor regional complexa. As características das lesões, os dados intraoperatórios e o acompanhamento ambulatorial estão detalhados na Tabela 1 .

**Tabela 1:** Características das lesões osteocondrais, dados intraoperatórios e acompanhamento

Paciente (idade em anos)	Topografia da fixação	Tempo da lesão à cirurgia (dias)	Tamanho da lesão (cm ^2^ )	N ^o^ de palitos ósseos	Cirurgia associada	Complicações	Acompanhamento (meses)
1 (19)	Patela	3	3.0	3	Não	Não	47
2 (19)	Patela	4	3.8	7	Não	Não	44
3 (18)	Patela	5	4	5	Não	Não	44
4 (20)	Patela	3	2.2	3	Não	Rigidez articular	44
5 (27)	CFL	2	4	6	Não	Não	43
6 (29)	Patela	2	3.5	6	RLPFM + RRL	Não	41
7 (25)	CFL	3	3	3	RLPFM	Rigidez articular/SDRC	30
8 (18)	Patela	5	2.5	3	Não	Não	24
9 (26)	Patela	3	2.5	3	Não	Não	21
10 (13)	Patela	4	2	3	Não	Não	18
11 (15)	Patela	4	0.8	2	RLPFM	Não	17

**Abreviaturas:** CFL, côndilo femoral lateral; RLPFM, reconstrução do ligamento patelofemoral medial; RRL, *release* do retináculo lateral; SDRC, síndrome de dor regional complexa.

**Fig. 4:**
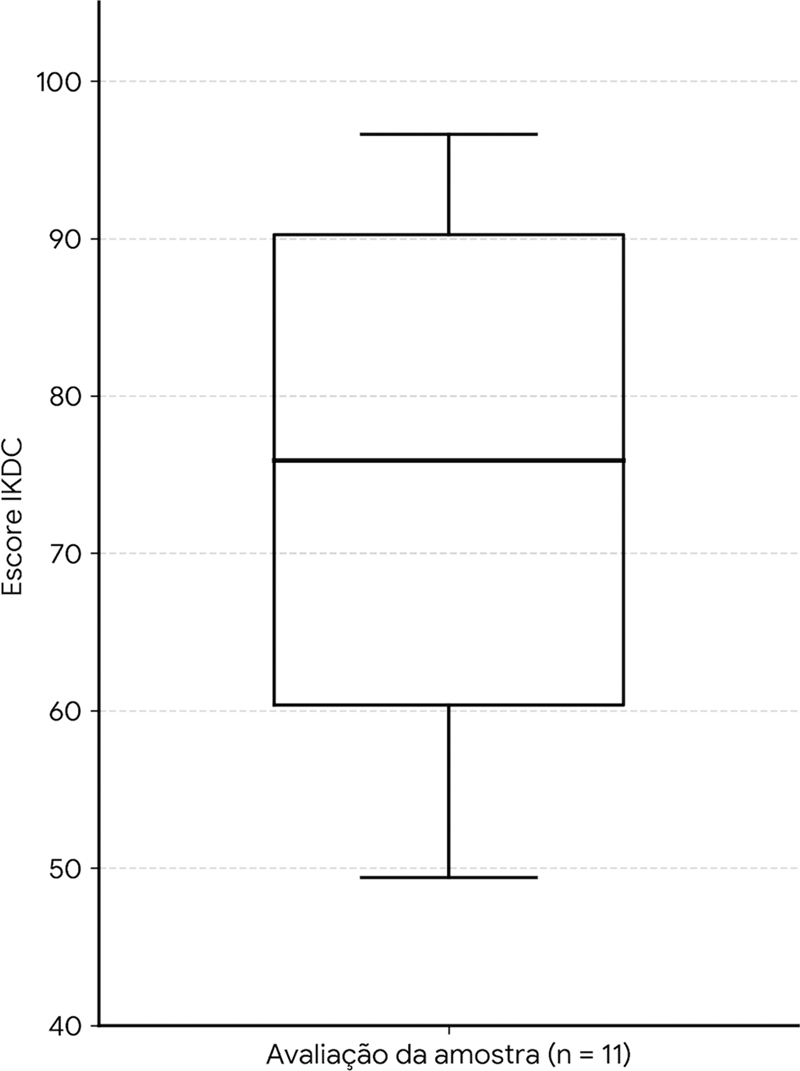
Diagrama de caixa da pontuação no Formulário de Avaliação Subjetiva do Joelho do International Knee Documentation Committee (IKDC).

Para a análise funcional dos pacientes, utilizou-se o formulário do IKDC, que abrange três domínios principais: sintomas, funcionalidade do joelho e nível de atividade esportiva. A média das pontuações obtidas foi de 75,0 ± 17,1, com mediana de 75,9 ( Fig. 4 ).

A avaliação funcional pelo formulário do IKDC demonstrou desfechos clinico-funcionais satisfatórios nos três domínios principais. No aspecto sintomático, a mediana da dor foi baixa (1,5 de 10), com predominância de relatos de rigidez ou inchaço ausentes ou leves. Em termos de funcionalidade geral, a pontuação média foi de 75,0 ± 17,1 (valor máximo de 96,0), o que sugere recuperação satisfatória na maioria dos casos. No domínio esportivo, mais da metade dos pacientes retomou atividades moderadas, e uma parcela relatou tolerância a exercícios vigorosos. Os dados detalhados estão apresentados no Anexo 1.

## Discussão

Apesar de descrita como uma alternativa biológica promissora, a fixação de lesões osteocondrais com palitos ósseos autólogos ainda é pouco explorada na literatura, sendo mais frequentemente relatada em casos de osteocondrite dissecante 10 19 20 ou em estudos isolados com relatos de fraturas osteocondrais tratadas por essa técnica. 7 8 Diante desse cenário, este estudo teve como objetivo descrever uma série de casos de fraturas osteocondrais traumáticas do joelho tratadas com palitos ósseos autólogos, com análise funcional ao longo de um acompanhamento de 3 anos.

Neste estudo, a maioria dos pacientes apresentou recuperação funcional satisfatória, com média de 75,0 ± 17,1 pontos no formulário IKDC e elevada taxa de retorno às atividades físicas de intensidade moderada ou vigorosa. A percepção subjetiva de funcionalidade do joelho manteve-se estável, com médias de 8,8 ± 1,3 pontos no período pré-lesão, e de 8,6 ± 1,4 no pós-operatório, e mediana de 9,0 em ambos os momentos. Esses resultados são comparáveis aos descritos por Ogura et al., 2 que relataram taxa de sucesso de 83% em 6 adolescentes (média etária de 12,9 anos) tratados com fixação de fragmentos condrais puros por palitos ósseos autólogos, acompanhados por uma média de 5,2 (variação: 1,4–10,9) anos. Nesse estudo, os pacientes apresentaram retorno pleno ao esporte em média aos 7 meses e pontuação média de 85 (variação: 70–95) no sistema Magnetic Resonance Observation of Cartilage Repair Tissue (MOCART), o que evidencia cicatrização condral adequada na RM. Apesar das diferenças populacionais, ambos os trabalhos demonstram recuperação funcional satisfatória, preservação da congruência articular e bom potencial biológico de integração osteocondral proporcionado pela fixação com palitos ósseos autólogos.

Os resultados positivos observados neste estudo podem estar associados ao perfil etário da amostra. Os pacientes incluídos eram jovens, com média de 20,3 anos, faixa etária na qual se espera melhor prognóstico em casos de lesões condrais. Em estudo prévio 21 realizado por nosso grupo, identificamos que a idade constitui um dos principais fatores prognósticos em intervenções para lesões osteocondrais, sendo que pacientes com mais de 40 anos tendem a apresentar piores desfechos clínicos e funcionais. Assim, o bom desempenho funcional observado nesta série pode refletir também a capacidade biológica superior de integração de indivíduos jovens.

Entre os pacientes incluídos neste estudo, foram observadas duas complicações pós-operatórias: 2 pacientes apresentaram rigidez articular, uma delas associada à síndrome de dor regional complexa (SDRC). Em ambos os casos, optou-se pela realização de manipulação articular sob anestesia/sedação. Em seguida, ambas foram submetidas a um protocolo intensivo de fisioterapia e fortalecimento muscular, e evoluíram satisfatoriamente, sem necessidade de novas intervenções. A SDRC foi manejada clinicamente com o uso de analgésicos e moduladores de dor. Não foram registrados casos de infecção, formação de corpos livres ou necessidade de remoção dos palitos ósseos.

Sob o ponto de vista biomecânico, o estudo experimental de Sasaki et al. 22 comparou três métodos de fixação em modelo animal – palitos ósseos autólogos, pinos bioabsorvíveis e âncoras de sutura com fita 2-0. Demonstrou-se que os palitos apresentaram resistência inicial compatível com as demandas do ambiente intra-articular, embora inferior à observada no grupo das âncoras. É importante ressaltar que não houve diferença estatisticamente significativa entre os grupos quanto à carga necessária para provocar deslocamento de 1 mm, parâmetro crítico de estabilidade primária. Esses achados experimentais são consistentes com os resultados observados neste estudo, em que a técnica demonstrou estabilidade adequada e ausência de falhas mecânicas durante o acompanhamento.

Em comparação com outras técnicas de fixação, a sutura transóssea representa uma alternativa que também evita o uso de implantes proeminentes. Estudos que empregaram essa abordagem no tratamento das fraturas osteocondrais patelares relataram bons resultados clínicos e altas taxas de união. 23 24 Do ponto de vista biomecânico, a principal vantagem dos palitos ósseos autólogos em relação à sutura pode residir na maior estabilidade compressiva e rotacional do fragmento, particularmente em lesões de maior dimensão ou com osso subcondral mais espesso. Por outro lado, a sutura transóssea pode ser preferível em fragmentos com osso subcondral mais fino, nos quais a inserção de palitos ósseos se torna tecnicamente mais desafiadora. Dessa forma, a escolha entre as técnicas de fixação deve ser individualizada, considerando as características do fragmento osteocondral, a qualidade do osso subcondral e a experiência do cirurgião.

Apesar dos bons resultados descritos com o uso de implantes metálicos e dispositivos bioabsorvíveis na fixação de fraturas osteocondrais, 4 13 25 essas técnicas não estão isentas de complicações. Entre as principais desvantagens relatadas estão a necessidade de reintervenção cirúrgica para a retirada do material, o risco de comprometimento da cartilagem adjacente, reações inflamatórias relacionadas ao material de síntese, e a possibilidade de má consolidação do fragmento osteocondral. 1 4 12 13 14 16 25 Em contraste, a fixação com palitos ósseos autólogos elimina o risco de resposta inflamatória ao implante, dispensa retirada cirúrgica e preserva a integridade da cartilagem, de modo que representa uma alternativa biológica e economicamente viável.

Apesar das vantagens biológicas associadas à técnica, algumas limitações operatórias devem ser consideradas. A confecção dos palitos ósseos requer a retirada de um segmento da metáfise proximal da tíbia, o que pode resultar em dor local, hematoma e, raramente, fratura. 19 Trata-se de um procedimento com elevada dependência técnica, que exige precisão tanto na modelagem dos enxertos quanto no posicionamento intra-articular, sob risco de instabilidade do fragmento ou incongruência da superfície articular.

**Fig. 5:**
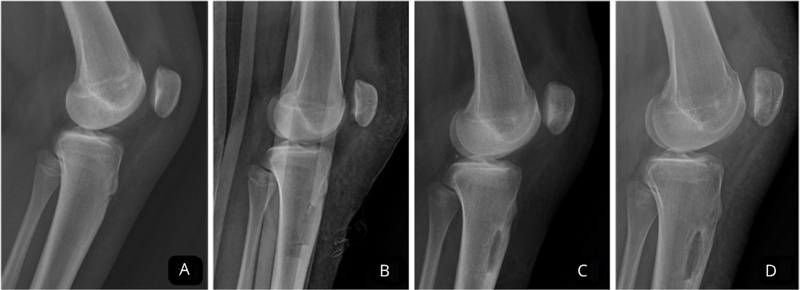
Evolução radiográfica evidenciando consolidação em lesão osteocondral patelar. ( **A** ) Radiografia admissional; ( **B** ) pós-operatório imediato (em uso de *brace* ); ( **C** ) 3 meses de evolução; e ( **D** ) 6 meses de evolução.

Nesta série, observou-se evidência clínica e radiográfica de integração óssea satisfatória em todos os casos ( Fig. 5 ), que geralmente ocorreu entre 3 e 6 meses do acompanhamento pós-operatório, sem registro de falhas de consolidação ou necessidade de reintervenções. Embora nem todos os pacientes tenham realizado RM de controle ou tenham sido submetidos à aplicação de escores específicos como o MOCART, 26 os dados disponíveis sugerem adequada incorporação do fragmento osteocondral. Em três casos, foram identificadas áreas de esclerose subcondral nos exames de imagem, achado previamente descrito como possível consequência de técnicas que envolvem perfuração do osso subcondral para a inserção de implantes. 2 27 28 Até o último acompanhamento, essas alterações não demonstraram repercussão clínica. Ainda assim, destaca-se a importância do acompanhamento clínico e radiológico prolongado, considerando que alterações subcondrais podem, em outros contextos, estar associadas à falha de integração ou degeneração cartilaginosa tardia. 26

Entre as limitações deste estudo, destaca-se, primeiramente, o delineamento como série de casos, o que restringe a generalização dos achados. A falta de coleta sistemática da pontuação pré-operatória no formulário do IKDC nos impede de quantificar a melhora funcional de forma objetiva. Além disso, embora todos os pacientes tenham apresentado consolidação radiográfica satisfatória, nem todos foram submetidos a exames de imagem avançados, como a RM, o que inviabiliza a aplicação sistemática de escores específicos, como o MOCART. 26 A ausência de um grupo controle tratado com outra técnica de fixação também limita comparações diretas entre diferentes abordagens. O tempo de acompanhamento, embora adequado para a avaliação funcional inicial, ainda é insuficiente para detectar possíveis alterações subcondrais tardias. Estudos futuros, com maior número de pacientes, acompanhamento prolongado e avaliações funcional e radiológica padronizadas, são fundamentais para validar e expandir os achados desta investigação.

## Conclusão

A fixação de fraturas osteocondrais do joelho com palitos ósseos autólogos demonstrou ser uma técnica eficaz, que resultou em consolidação óssea consistente e desfechos funcionais favoráveis nesta série de casos. O procedimento representa uma alternativa biológica e de baixo custo, que evita complicações associadas a implantes metálicos ou bioabsorvíveis. Os achados sugerem que essa técnica é uma opção de tratamento viável e segura para pacientes jovens com esse tipo de lesão.
